# Relationship Between Tamsulosin Use and Surgical Complications of Cataract Surgery in Elderly Patients: Population-Based Cohort Study

**DOI:** 10.3389/fmed.2022.882131

**Published:** 2022-05-19

**Authors:** Jiehoon Kwak, Jung Yeob Han, Su Young Moon, Sanghyu Nam, Jae Yong Kim, Hungwon Tchah, Hun Lee

**Affiliations:** Department of Ophthalmology, Asan Medical Center, University of Ulsan College of Medicine, Seoul, South Korea

**Keywords:** cataract surgery, tamsulosin, surgical complication, KNHIS-Senior cohort, cataract (senile)

## Abstract

**Purpose:**

Although several previous studies have investigated the relationship between tamsulosin use and surgical complications of cataract surgery, no population-based cohort study has been conducted for the Asian population. We aimed to investigate the relationship between tamsulosin use and surgical complications of cataract surgery in the Korean elderly population.

**Methods:**

This nationwide population-based retrospective cohort study included elderly patients (≥60 years) who had undergone cataract surgery in the period from 2003 to 2015. Baseline characteristics were age, sex, income, residence, and systemic, and ocular comorbidities (glaucoma, myopia, eye trauma, diabetes mellitus with ophthalmic manifestations, severe cataract, age-related macular degeneration). The exposure of interest was tamsulosin use within 1 year before cataract surgery. Logistic regression model was used to evaluate the relationship of tamsulosin use with surgical complications of cataract surgery.

**Results:**

The rate of surgical complications of cataract surgery was 0.88% (375/42,539) in the non-tamsulosin group and 0.83% (71/8,510) in the tamsulosin group. The groups showed no significant difference in the risk of surgical complications of cataract surgery in the unadjusted model [odds ratio (OR) = 0.946; 95% confidence interval (CI):0.733–1.220; *P* = 0.669]. Additionally, tamsulosin use was not significantly associated with surgical complications of cataract surgery in the fully adjusted model accounting for age, income, residence, and systemic and ocular comorbidities (OR = 0.997; 95% CI: 0.749–1.325; *P* = 0.981).

**Conclusions:**

The rate or risk of surgical complications of cataract surgery does not change with tamsulosin use. We suggest that better surgical techniques and surgeons' cognizance of the patient's tamsulosin use could improve surgical outcomes, without increasing surgical complications.

## Introduction

Tamsulosin is a subtype-selective alpha (1A and 1D) adrenoceptor antagonist that induces relaxation of smooth muscles in the prostate and bladder (1). It is commonly used to treat symptomatic benign prostate hyperplasia (BPH) and kidney stones and was approved for use in the United States in 1997 and in South Korea in 2006. It has been prescribed globally to treat acute urinary retention caused by BPH, and systemic side effects, e.g., hypotension, have been relatively uncommon (1).

Intraoperative floppy iris syndrome (IFIS), which comprises intraoperative progressive miosis, iris prolapse, and iris billowing, is frequent in patients taking tamsulosin (2–4). The prevalence of IFIS is 2%, and most cases are related to tamsulosin use (5, 6). In addition, patients who are administered with tamsulosin preoperatively tend to develop miotic pupils, iris prolapse at the incision margin, and hypotonic iris during cataract surgery (2, 7, 8). Such anatomical or functional changes induced by tamsulosin increase the difficulty of cataract surgery, which could lead to perioperative or postoperative complications (2, 8, 9). Nonetheless, owing to the surgeon's effort to respond to the risk posed by tamsulosin use and advances in surgical equipment, surgical outcomes of cataract surgery improved in patients taking tamsulosin (9–12).

Previous studies evaluated surgical complications of cataract surgery associated with tamsulosin use (2, 6). A recent population-based study on yearly cataract surgical complication rates of patients taking tamsulosin demonstrated that the risk of cataract surgical complications decreased with time with or without tamsulosin use (13). However, it is unclear whether those findings are generalizable to the Korean elderly population, and no population-based cohort study has been conducted for the Asian population. Therefore, the aim of the present study was to investigate the relationship between tamsulosin use and surgical complications of cataract surgery in the Korean elderly population using the Korean National Health Insurance Service-Senior cohort (NHIS-Senior) database.

## Methods

### Study Design and Data Source

This was a population-based retrospective cohort study conducted using the KNHIS-Senior database. The health insurance system in South Korea is a nationwide universal single-payer system managed by the KNHIS. The KNHIS-Senior database is provided by the KNHIS. It includes the data of 558,147 individuals randomly sampled from 10% of the approximate 5.5 million South Korean people aged ≥60 years, including information on age, sex, general health examinations, hospital and pharmacy visits, disease diagnoses and status, procedures, and prescribed medications (14, 15). All participants included in the NHIS-Senior database were followed-up until 2015 unless they were disqualified for health coverage reasons, such as death or emigration.

The KNHIS uses Korean Electronic Data Interchange (KEDI) and Korean Standard Classification of Diseases (KCD) codes, a system similar to the International Classification of Diseases (16). As the NHIS-Senior database comprises publicly accessible data, the Institutional Review Board of Asan Medical Center (University of Ulsan College of Medicine) instead of approved the waiver of reviewing this study (2020-1194). This study was conducted according to the ethical principles outlined in the Declaration of Helsinki. The requirement for obtaining informed consent was waived because anonymized and de-identified data were used for analyses.

### Study Population

We selected the target population among those who were included in the NHIS-Senior database from 1 January 2002 to 31 December 2015 (*n* = 558,147). Initially, we applied the wash-out period of between 1 January and 31 December 2002 to reduce the potential risk of surveillance bias. The inclusion criterion was the presence of at least one NHIS record from 1 January 2003 to 15 December 2015 with the following conditions (*n* = 54,236): a KEDI code for cataract surgery and men aged ≥60 years in this period. Eligible subjects were classified into tamsulosin and non-tamsulosin groups according to tamsulosin use within 1 year before cataract surgery. Patients with the following characteristics were excluded: age < 60 years; procedures combined with vitrectomy or glaucoma surgery; and prior ocular procedures, including intraocular surgery or intravitreal injections within 1 year before cataract surgery; or retinal laser procedures within 5 years before cataract surgery. Patients who underwent simultaneous bilateral cataract surgery were excluded to avoid confounding.

The exposure of interest was tamsulosin use within 1 year before cataract surgery, except for tamsulosin medication on the same day. The tamsulosin group (*n* = 8,510) comprised participants with a KEDI code for cataract surgery and tamsulosin use within 1 year before cataract surgery. For each patient, cataract surgery was defined as the simultaneous claim of extracapsular or intracapsular extraction (KEDI code: S5111) or phacoemulsification (KEDI code: S5119) and primary intraocular lens implantation (KEDI code: S5117) on the same day (KEDI codes: S5111 + S5117 and S5119 + S5117). The non-tamsulosin group (*n* = 42,539) comprised participants with a KEDI code for cataract surgery but without tamsulosin use within 1 year before cataract surgery. Additionally, to investigate relationship between the use of alpha antagonist and surgical complications of cataract surgery in the Korean elderly population, we performed the comparison analysis between alpha antagonist (terazosin, alfuzosin, doxazosin, silodosin, and tamsulosin) group and non-alpha antagonist group.

### Surgical Complication Events as the Outcome Measure

Surgical complications of cataract surgery, which is associated with tamsulosin use, included posterior capsule rupture (PCR), dropped lens fragments, retinal detachment, and suspected endophthalmitis (13). The use of anterior vitrectomy for intraoperative PCR was documented if the KEDI code S5122 was reported between the cataract surgery day and 2 weeks after cataract surgery. The use of total vitrectomy for intraoperative PCR, dropped lens fragments, or suspected endophthalmitis was documented if the KEDI code S5121 was reported between 1 day and 2 weeks after cataract surgery. Similarly, the use of retinal detachment operation for retinal detachment was documented if the KEDI code S5130 was reported between 1 day and 2 weeks after cataract surgery. Even though KEDI codes S5122 and S5122 have been also indicated in other vitreoretinal diseases, we made an assumption that vitrectomy within 2 weeks after cataract surgery is highly related with secondary surgery which is associated with surgical complication from cataract surgery. Only the first event was included in cases of multiple complications.

### Covariates

Demographics included age at the time of cataract surgery, sex, residence, and income level; the residential area was divided into metropolitan (Seoul and large cities) and provincial regions (small cities and rural areas) according to the administrative unit of Korea. Household income was categorized as below or above 20% of the income. Both systemic and ocular comorbidities were included as covariates and assessed at the time of cataract surgery. Systemic comorbidities included diabetes (KCD codes: E10–E14), hypertension (KCD codes: I10–I15), BPH (KCD code: N40), and vascular disease (KCD codes: I20–I25, I61, I63–I66, I67.2, I67.8, I69, I70, I73, and I74). Ocular comorbidities included glaucoma (KCD codes: H40 and H42), myopia (KCD codes: H52.1 and H44), eye trauma (KCD code: S05), diabetes mellitus (DM) with ophthalmic manifestations (KCD codes: E10.3, E11.3, E12.3, E13.3, and E14.3), severe cataract (KCD codes: H25.2 and H25.1), and age-related macular degeneration (KCD codes: H35.30, H35.31, and H35.39). The presence of severe cataract was recognized as an indicator of poor vision because visual acuity data were not available (17, 18). Patients with diagnostic codes for brunescent cataract and morgagnian cataract were considered to have severe cataract (17, 18).

### Statistical Analysis

A logistic regression model was used to evaluate the relationship between tamsulosin use and surgical complications of cataract surgery. We used two models of adjustment to account for potential confounding factors. Model 1 was adjusted for age (<70, 70–80, 80–90, and ≥90 years). Model 2 was further adjusted for income, residence, and systemic and ocular comorbidities. All statistical analyses were conducted using SAS software, version 9.4 (SAS Institute, Cary, NC, United States). Statistical significance was considered at a two-sided *p*-value < 0.05. The absolute standardized difference (ASD) was used to compare baseline characteristics. An ASD > 0.1 was considered to be clinically meaningful (19).

## Results

Table 1 summarizes baseline characteristics. The study cohort included 51,049 patients, 42,539 of whom were in the non-tamsulosin group whereas 8,510 in the tamsulosin group. The largest proportion of patients in both groups was 70–80 years old at the time of cataract surgery (61.69 and 65.22%). Compared to patients in the non-tamsulosin group, those in the tamsulosin group were slightly older (ASD = 0.3289) and had a significantly higher proportion of systemic diseases, such as diabetes, hypertension, and vascular disease (ASD = 0.2814, 0.2888, and 0.3967, respectively). In terms of ocular comorbidity, the tamsulosin group had a higher proportion of age-related macular degeneration (ASD = 0.1115). The development of surgical complications of cataract surgery did not differ significantly between tamsulosin and non-tamsulosin groups [71 (0.83%) cases in the tamsulosin group and 375 (0.88%) cases in the non-tamsulosin group; ASD = 0.0051]. In addition, the rate of surgical complications of cataract surgery did not differ between alpha antagonist (terazosin, alfuzosin, doxazosin, silodosin, and tamsulosin) group and non-alpha antagonist group (ASD = 0.0174; Supplementary Table 1).

**Table 1:** Baseline characteristics of subjects who underwent cataract surgery according to tamsulosin use in the Korean elderly population.

**Variable**	**Non-tamsulosin group (*n* = 42,539)**	**Tamsulosin group (*n* = 8,510)**	***P*-value**	**ASD^**a**^**
Age (years)				0.3289
<70	8,348 (19.62)	760 (8.93)	<0.001	
70–80	26,242 (61.69)	5,550 (65.22)	<0.001	
80–90	7,541 (17.73)	2,103 (24.71)	<0.001	
≥90	408 (0.96)	97 (1.14)	<0.001	
Mean ± SD	74.44 ± 5.78	76.19 ± 5.34	<0.001	0.3160
Residence			0.047	0.0235
Metropolitan	16,798 (39.48)	3,458 (40.63)		
Provincial	25,741 (60.51)	5,052 (59.36)		
Income			0.140	0.0175
Below 20 percentiles	8,568 (20.14)	1,774 (20.84)		
Above 20 percentiles	33,971 (79.86)	6,736 (79.15)		
Diabetes	23,002 (54.07)	5,759 (67.67)	<0.001	0.2814
Hypertension	29,911 (70.31)	7,017 (82.46)	<0.001	0.2888
BPH	16,989 (39.94)	8,410 (98.82)	<0.001	1.6607
Vascular disease	24,931 (58.61)	6,536 (76.8)	<0.001	0.3967
Glaucoma	280 (0.66)	93 (1.09)	<0.001	0.0467
Myopia	6,470 (15.21)	1,513 (17.78)	<0.001	0.0693
Eye trauma	1,537 (3.61)	375 (4.41)	<0.001	0.0404
DM with ophthalmic manifestations	1,299 (3.05)	350 (4.11)	<0.001	0.0570
Severe cataract	14,513 (34.12)	3,186 (37.44)	<0.001	0.0693
Age-related macular degeneration	1,541 (3.62)	511 (6.0)	<0.001	0.1115
Event of complication	375 (0.88)	71 (0.83)	0.669	0.0051

*SD, standard deviation; BPH, Benign prostatic hyperplasia; DM, diabetes mellitus*.

*Data are expressed as the mean ± SD, or n (%)*.

*^a^ASD of > 0.1 is considered meaningful imbalances*.

Table 2 shows the odds ratio (OR) of surgical complications in the Korean elderly population with cataract surgery according to tamsulosin use. There was no significant difference in OR of surgical complication events between tamsulosin and non-tamsulosin groups in the unadjusted model [OR = 0.946; 95% confidence interval (CI): 0.733–1.220; *P* = 0.669]. Even after adjusting for age, OR of surgical complication events did not differ significantly between tamsulosin and non-tamsulosin groups (OR = 0.950; 95% CI: 0.735–1.228; *P* = 0.694). Additionally, tamsulosin use was not significantly associated with surgical complications of cataract surgery in the fully adjusted model accounting for age, income, residence, systemic and ocular comorbidities (OR = 0.997; 95% CI: 0.749–1.325; *P* = 0.981). Furthermore, the use of alpha antagonist was not associated with surgical complications of cataract surgery in the fully adjusted model accounting for age, income, residence, systemic and ocular comorbidities (OR = 0.813; 95% CI: 0.624–1.059; *P* = 0.130; Supplementary Table 2).

**Table 2:** Odds Ratio of complication event of cataract surgery in the Korean elderly population with cataract surgery according to tamsulosin use.

**Non-tamsulosin vs. tamsulosin**	**Odds ratio**	**95% CI**	***P*-value**
Crude (no adjustment)	0.946	0.733–1.220	0.669
Adjusted for age	0.950	0.735–1.228	0.694
Adjusted for age, income, residence, systemic, and ocular comorbidities^a^	0.997	0.749–1.325	0.981

*CI, confidence interval*.

*^a^Diabetes, hypertension, benign prostatic hyperplasia, vascular disease, glaucoma, myopia, eye trauma, DM with ophthalmic manifestations, severe cataract, and age-related macular degeneration*.

In the tamsulosin group, myopia was associated with decreased surgical complications of cataract surgery (OR = 0.419; 95% CI: 0.180–0.971; *P* = 0.043; Table 3). Both eye trauma (OR = 1.849; 95% CI: 1.215–2.814; *P* = 0.004) and severe cataract (OR = 1.335; 95% CI: 1.083–1.645; *P* = 0.007) were associated with increased surgical complications of cataract surgery in the non-tamsulosin group (Table 4).

**Table 3:** Effects of Calendar Year and Covariates on complication event of cataract surgery in the tamsulosin group.

**Tamsulosin group (*n* = 8,510)**	**Odds ratio**	**95% CI**	***P*-value**
Calendar year (per additional year)	0.985	0.907–1.07	0.719
Patient–level effects (vs. age <70)			
Age 70–80 yrs	1.556	0.538–4.502	0.415
Age 80–90 yrs	1.653	0.535–5.105	0.382
Age ≥ 90 yrs	3.805	0.661–21.891	0.135
Diabetes	1.270	0.739–2.185	0.387
Hypertension	0.869	0.459–1.645	0.667
Vascular disease	1.100	0.598–2.025	0.760
Glaucoma	<0.001		0.986
Myopia	0.419	0.180–0.971	0.043
Eye trauma	0.303	0.042–2.198	0.238
DM with ophthalmic manifestations	0.646	0.156–2.673	0.546
Severe cataract	1.115	0.689–1.803	0.659
Age–related macular degeneration	1.839	0.811–4.168	0.144

*CI, confidence interval; DM, diabetes mellitus*.

**Table 4:** Effects of Calendar Year and Covariates on complication event of cataract surgery in the non-tamsulosin group.

**Non-tamsulosin group (*n* = 42,539)**	**Odds ratio**	**95% CI**	***P*-value**
Calendar year (per additional year)	1.017	0.982–1.052	0.342
Patient-level effects (vs. age <70)			
Age 70–80 yrs	0.783	0.592–1.036	0.087
Age 80–90 yrs	0.938	0.667–1.318	0.712
Age ≥ 90 yrs	1.186	0.474–2.969	0.715
Diabetes	0.941	0.755–1.172	0.586
Hypertension	0.862	0.68–1.094	0.222
Vascular disease	0.967	0.766–1.221	0.778
Glaucoma	0.842	0.208–3.402	0.809
Myopia	0.786	0.577–1.069	0.124
Eye trauma	1.849	1.215–2.814	0.004
DM with ophthalmic manifestations	0.750	0.368–1.526	0.427
Severe cataract	1.335	1.083–1.645	0.007
Age-related macular degeneration	1.022	0.589–1.771	0.939

*CI, confidence interval; DM, diabetes mellitus*.

## Discussion

This nationwide population-based cohort study demonstrated that surgical complications of cataract surgery were not significantly affected by tamsulosin use, although there was a trend of increasing rate of surgical complications of cataract surgery after adjusting for demographics and systemic and ocular comorbidities. In patients with tamsulosin use within 1 year preceding cataract surgery, no specific risk factor was associated with the surgical complication event during cataract surgery.

IFIS and other surgical complications have been reported since the worldwide use of tamsulosin began for acute urinary retention in the old age group, which is the most common age group undergoing cataract surgery. Previous studies on surgical complications of cataract surgery in patients taking tamsulosin demonstrated that IFIS occurred in 2% of cataract surgeries, and adjunctive measures for pupil dilation were ineffective compared to non-tamsulosin users (5). Cataract surgical complications, including retinal detachment, loss of lens fragment, and endophthalmitis, were significantly more prevalent (OR = 2.33; CI: 1.22–4.43) in tamsulosin users (2). A previous study demonstrated that doxazosin (an alpha blocker for BPH) was related to higher risks of PCR and vitreous loss (OR = 1.51; CI: 1.09–2.07; adjusted model) (20). However, unlike those previous results, a recent large population study from Canada and our large cohort study showed that tamsulosin use was not associated with increased cataract surgical complications (13).

In a recently published population-based study, Campbell et al. showed that the risk of surgical complications of cataract surgery, such as PCR, dropped lens fragments, retinal detachment, and suspected endophthalmitis, significantly decreased with time from 2003 to 2013 in patients with tamsulosin use within 1 year preceding cataract surgery (OR = 0.95/year; 95% CI: 0.91–0.99/year; *P* = 0.010) (13). The risk also decreased in patients without tamsulosin use within 1 year preceding cataract surgery. However, those results did not reflect direct comparison results between patients with and without tamsulosin use. In our study, there was no significant difference in the risk of surgical complications of cataract surgery between tamsulosin and non-tamsulosin groups in the unadjusted model (OR = 0.946; 95% CI: 0.733–1.220; *P* = 0.669). In addition, tamsulosin use was not significantly associated with surgical complications of cataract surgery in the fully adjusted model accounting for age, income, residence, and systemic and ocular comorbidities (OR = 0.997; 95% CI: 0.749–1.325; *P* = 0.981). Nevertheless, surgeons should devote efforts to avoid adverse surgical events, including PCR and dropped lens fragments. Considering that IFIS increases surgical difficulties, which might lead to PCR and vitreous prolapse, we included partial anterior vitrectomy on the day of cataract surgery to manage such surgical complications, which can be considered to be our novelty.

After the introduction of IFIS caused by tamsulosin and cognizance of significant risks posed by tamsulosin use, several surgical techniques were introduced worldwide (10, 11, 21). Although our study lacks the usage of adjunctive measures, such as drugs or device during cataract surgery, we suggest that comparable results between tamsulosin and non-tamsulosin groups can be attributed to efforts to respond to the risk posed by tamsulosin use using sophisticated surgical instruments or an intraoperative epinephrine injection into the anterior chamber. Particularly, viscoadaptive ophthalmic viscosurgical devices, fluidic parameter optimization, mechanical pupil expansion devices, and intensive pharmacologic pupil dilation can be applied to increase the efficiency and safety of cataract surgery in patients with tamsulosin use (22–24). Recently introduced femtosecond laser-assisted cataract surgery (FLACS) can be helpful for safe cataract surgery in eyes with a small pupil due to tamsulosin use (25, 26). Conrad-Hengerer et al. demonstrated that surgically dilating small pupils before femtosecond laser using intracameral epinephrine, viscomydriasis, and pupil expander can assist safe anterior capsulotomy and nuclear fragmentation (25). Although our cohort data did not include FLACS as a variable in cataract surgery, such a cutting-edge technique can improve surgical outcomes. Moreover, surgeons' recognition of the perioperative risk during cataract surgery in patients with tamsulosin use could decrease the complication rate (9).

Our study included various variables related to cataract surgery: age, socioeconomic state, systemic disease (DM, hypertension, BPH, and vascular disease), and ocular comorbidities (glaucoma, myopia, eye trauma, DM with ophthalmic manifestations, severe cataract, and age-related macular degeneration). The presence of ocular trauma, severity of cataract, presence of myopia, and DM increase surgical complications of cataract surgery. Among them, ocular trauma is associated with anatomical deformities of the iris, zonules, and lens, which can subsequently lead to surgical difficulties during cataract surgery and increased surgical complications (20, 27). Similarly, severe cataract could make phacoemulsification difficult and be more vulnerable to surgical complications (20). Lacking of information in cataract grading with slit lamp examination which is based on the LOCS grading system is the limitation of our study. Thus, we hypothesized that including the severe cataract as covariates can be meaningful after defining the severe cataract using the KCD codes (H25.1 and H25.2) in order to investigate the effect of severity of cataract on the relationship between tamsulosin use and surgical complications of cataract surgery. However, in the tamsulosin group, no significant risk factor was associated with surgical complications of cataract surgery. Protective association was noted between surgical complications and myopia in the tamsulosin group. In contrast, in the non-tamsulosin group, both eye trauma and severe cataract were associated with surgical complications of cataract surgery. We assumed that surgeons' recognition of possible intraoperative complications related to tamsulosin use can lead to use careful surgical maneuver and supportive techniques, which eventually decrease surgical complications in patients with ocular trauma and severe cataract.

Our study had some limitations. First, this study was mainly limited by its observational nature. Second, as this study was based on data from a medical insurance claims database, identification of patients with cataract surgery and diagnostic accuracy of systemic and ocular comorbidities might be inaccurate compared to information obtained from medical charts. Moreover, the NHIS-Senior database cannot provide information on cataract grading, objective visual acuity, axial length, presence of pseudoexfoliation syndrome, or postoperative inflammation grade. In addition, there was a lack of availability of certain covariates including surgeons' experience, metabolic profiles, body mass index, alcohol intake, smoking status, and physical activity, thereby proposing the need for further studies including various covariates. Third, among various types of alpha antagonist, including tamsulosin, terazosin, silodosin, doxazosin, etc., only the use of tamsulosin was included in the current study because the tamsulosin is the most commonly prescribed medication among them for treating BPH in South Korea (Supplementary Table 1). When interpreting the results, clinicians should be in cautious since it may lead to a bias and overall results cannot be generalized to patients taking medication other than tamsulosin. Nevertheless, the rate of surgical complications of cataract surgery did not differ between alpha antagonist group and non-alpha antagonist group (Supplementary Tables 1–4). Fourth, the primary outcome of surgical complications was defined as secondary vitrectomy surgery within 14 postoperative days. The NHIS-Senior database lacks information on cataract surgery-related minor anatomical and/or functional complications, such as iris prolapse, iris atrophy, and pupil abnormality. Therefore, our study had a limitation of overlooking cataract surgery-related minor anatomical and/or functional complications, which are not indications for the secondary operation. Therefore, overall surgical complication rates (0.87%) might have been underestimated. Delayed onset complications, such as delayed endophthalmitis and intraocular lens dislocation, were possibly excluded. Additionally, glaucoma filtering surgery due to increased intraocular pressure or intraocular lens sulcus insertion due to posterior capsular rupture might be possibly excluded. We counted the first adverse event after cataract surgery, so additional surgical procedures for complications might have been missed. Finally, we focused only on South Korean residents. Therefore, observed findings cannot be generalized to other ethnic groups.

Within these limitations, this is the first report on the relationship between oral tamsulosin use and surgical complications of cataract surgery in the elderly South Korean patients using a nationwide, general population-based database. Moreover, this study used a large sample size of the NHIS-Senior database. Selection bias was relatively low because the entire Korean population was enrolled in the same insurance system.

In summary, despite concerns regarding perioperative and postoperative complications in cataract surgery related to tamsulosin use, our study demonstrated no statistically significant difference in surgical complication events of cataract surgery between tamsulosin and non-tamsulosin groups. Better techniques to manage a difficult cataract surgery and surgeons' cognizance of tamsulosin use could improve surgical outcomes.

## Data Availability Statement

The datasets presented in this article are not readily available. The data that support the findings of this study are available from NHIS, but restrictions apply to the availability of these data, which were used under license for the current study, and so are not publicly available. Data are however available from the authors upon reasonable request and with permission of NHIS. Requests to access the datasets should be directed to yhun777@gmail.com.

## Ethics Statement

The studies involving human participants were reviewed and approved by Institutional Review Board of Asan Medical Center and University of Ulsan College of Medicine approved the waiver of reviewing this study (2020-1194). Written informed consent for participation was not required for this study in accordance with the national legislation and the institutional requirements.

## Author Contributions

Conceptualization, formal analysis, investigation, writing—original draft preparation, and writing—review and editing: JKw, JH, SM, SN, JKi, HT, and HL. Methodology and data curation: JKw, JKi, HT, and HL. Supervision: JKi and HL. Project ad-ministration: JKi, HT, and HL. Funding acquisition: HL. All authors have read and agreed to the published version of the manuscript. All authors contributed to the article and approved the submitted version.

## Funding

This research was supported by the Korea Medical Device Development Fund grant funded by the Korea government (the Ministry of Science and ICT, the Ministry of Trade, Industry and Energy, the Ministry of Health & Welfare, the Ministry of Food and Drug Safety) (Project Number: 9991006821, KMDF_PR_20200901_0148), by Korean Fund for Regenerative Medicine funded by Ministry of Science and ICT, and Ministry of Health and Welfare (21C0723L1-11, Republic of Korea), and by a grant from the Asan Institute for Life Sciences, Asan Medical Center, Seoul, Korea (2021IP0061-2, 2022IP0019-1). The funding agencies had no role in the design or conduct of this study; the collection, management, analysis, or interpretation of the data; preparation, review, or approval of the manuscript; or in the decision to submit the manuscript for publication.

## Conflict of Interest

The authors declare that the research was conducted in the absence of any commercial or financial relationships that could be construed as a potential conflict of interest.

## Publisher's Note

All claims expressed in this article are solely those of the authors and do not necessarily represent those of their affiliated organizations, or those of the publisher, the editors and the reviewers. Any product that may be evaluated in this article, or claim that may be made by its manufacturer, is not guaranteed or endorsed by the publisher.
