# Supplementary material for: Relationship Between Tamsulosin Use and Surgical Complications of Cataract Surgery in Elderly Patients: Population-Based Cohort Study
Source: Front Med (Lausanne). 2022 May 19;9:882131. doi: 10.3389/fmed.2022.882131 (PMC9160597; doi:10.3389/fmed.2022.882131)
Supplement: Supplementary file 1 [file Data_Sheet_1.docx]

**Supplementary Table 1.** Baseline characteristics of subjects who underwent cataract surgery according to the use of alpha antagonist in the Korean elderly population

| Variable | Non-alpha antagonist  group  (n=42,539) | alpha antagonist group  (n=15,474) | *P*-value | ASD^a^ |
| --- | --- | --- | --- | --- |
| Age (years) |  |  |  | 0.3069 |
| <70 | 7,462 (21.0) | 1,646 (10.6) | <0.001 |  |
| 70-80 | 21,781 (61.2) | 10,011 (64.7) | <0.001 |  |
| 80-90 | 5,999 (16.9) | 3,645 (23.6) | <0.001 |  |
| ≥90 | 333 (0.9) | 172 (1.1) | <0.001 |  |
| Mean ± SD | 74.0 ± 5.8 | 76.0 ± 5.4 | <0.001 | 0.3066 |
| Residence |  |  | 0.456 | 0.0272 |
| Metropolitan | 14,076 (39.6) | 6,177 (39.9) |  |  |
| Provincial | 21,499 (60.4) | 9,297 (60.1) |  |  |
| Income |  |  | 0.137 | 0.0143 |
| Below 20 percentiles | 7,145 (20.1) | 3,197 (20.7) |  |  |
| Above 20 percentiles | 28,430 (79.9) | 12,277 (79.3) |  |  |
| Diabetes | 18,535 (52.1) | 10,226 (66.1) | <0.001 | 0.2873 |
| Hypertension | 24,317 (68.4) | 12,611 (81.5) | <0.001 | 0.3068 |
| BPH | 10,316 (29.0) | 15,083 (97.5) | <0.001 | 2.0169 |
| Vascular disease | 19,985 (56.2) | 11,482 (74.2) | <0.001 | 0.3853 |
| Glaucoma | 207 (0.6) | 166 (1.1) | <0.001 | 0.0542 |
| Myopia | 5,257 (14.8) | 2,726 (17.6) | <0.001 | 0.0771 |
| Eye trauma | 1,262 (3.5) | 650 (4.2) | <0.001 | 0.0339 |
| DM with ophthalmic manifestations | 999 (2.8) | 650 (4.2) | <0.001 | 0.0758 |
| Severe cataract | 11,926 (33.5) | 5,773 (37.3) | <0.001 | 0.0792 |
| Age-related macular degeneration | 1,248 (3.5) | 804 (5.2) | <0.001 | 0.0828 |
| Event of complication | 328 (0.9) | 118 (0.8) | 0.075 | 0.0174 |
| The proportion of prescribed alpha antagonist |  |  |  |  |
| terazosin |  | 4,780 (30.9) |  |  |
| alfuzosin |  | 2,456 (15.9) |  |  |
| doxazosin |  | 4,121 (26.6) |  |  |
| silodosin |  | 1,264 (8.2) |  |  |
| tamsulosin |  | 8,510 (55.0) |  |  |

SD, standard deviation; BPH, Benign prostatic hyperplasia; DM, diabetes mellitus.

Data are expressed as the mean ± SD, or n (%).

^a^ASD of > 0.1 is considered meaningful imbalances.

**Supplementary Table 2**. Odds Ratio of complication event of cataract surgery in the Korean elderly population with cataract surgery according to the use of alpha antagonist

| Non-alpha antagonist vs alpha antagonist | Odds ratio | 95% CI | *P*-value |
| --- | --- | --- | --- |
| Crude (no adjustment) | 0.826 | 0.669-1.020 | 0.080 |
| Adjusted for age | 0.827 | 0.668-1.023 | 0.080 |
| Adjusted for age, income, residence, systemic and ocular comorbidities^a^ | 0.813 | 0.624-1.059 | 0.130 |

CI, confidence interval.

^a^Diabetes, hypertension, benign prostatic hyperplasia, vascular disease, glaucoma, myopia, eye trauma, DM with ophthalmic manifestations, severe cataract, and age-related macular degeneration.

**Supplementary Table 3.** Effects of Calendar Year and Covariates on complication event of cataract surgery in the alpha antagonist group

| Alpha antagonist group (n=15,474) | Odds ratio | 95% CI | *P*-value |
| --- | --- | --- | --- |
| Calendar year (per additional year) | 1.024 | 0.961-1.091 | 0.467 |
| Patient-level effects (vs age < 70) |  |  |  |
| Age 70-80 yrs | 1.140 | 0.551-2.360 | 0.724 |
| Age 80-90 yrs | 1.412 | 0.649-3.071 | 0.384 |
| Age ≥ 90 yrs | 1.786 | 0.375-8.500 | 0.466 |
| Diabetes | 1.135 | 0.751-1.715 | 0.549 |
| Hypertension | 0.951 | 0.576-1.570 | 0.844 |
| Vascular disease | 1.206 | 0.749-1.942 | 0.440 |
| Glaucoma | <0.001 |  | 0.988 |
| Myopia | 0.521 | 0.286-0.950 | 0.033 |
| Eye trauma | 1.182 | 0.515-2.710 | 0.693 |
| DM with ophthalmic manifestations | 0.569 | 0.179-1.810 | 0.340 |
| Severe cataract | 0.979 | 0.672-1.426 | 0.911 |
| Age-related macular degeneration | 1.218 | 0.580-2.557 | 0.602 |

CI, confidence interval; DM, diabetes mellitus.

**Supplementary Table 4.** Effects of Calendar Year and Covariates on complication event of cataract surgery in the non-alpha antagonist group

| Non-alpha antagonist group (n=42,539) | Odds ratio | 95% CI | *P*-value |
| --- | --- | --- | --- |
| Calendar year (per additional year) | 1.005 | 0.968-1.042 | 0.808 |
| Patient-level effects (vs age < 70) |  |  |  |
| Age 70-80 yrs | 0.802 | 0.598-1.074 | 0.138 |
| Age 80-90 yrs | 0.902 | 0.626-1.300 | 0.580 |
| Age ≥ 90 yrs | 1.401 | 0.557-3.521 | 0.473 |
| Diabetes | 0.936 | 0.740-1.184 | 0.584 |
| Hypertension | 0.855 | 0.666-1.099 | 0.222 |
| Vascular disease | 0.938 | 0.732-1.202 | 0.614 |
| Glaucoma | 1.099 | 0.271-4.454 | 0.895 |
| Myopia | 0.804 | 0.578-1.117 | 0.193 |
| Eye trauma | 1.711 | 1.069-2.740 | 0.025 |
| DM with ophthalmic manifestations | 0.821 | 0.383-1.757 | 0.611 |
| Severe cataract | 1.434 | 1.147-1.792 | 0.002 |
| Age-related macular degeneration | 1.160 | 0.653-2.059 | 0.613 |

CI, confidence interval; DM, diabetes mellitus.
